# Supplementary material for: Mechanisms governing the pioneering and redistribution capabilities of the non-classical pioneer PU.1
Source: Nat Commun. 2020 Jan 21;11:402. doi: 10.1038/s41467-019-13960-2 (PMC6972792; doi:10.1038/s41467-019-13960-2)
Supplement: Supplementary file 7 — Source data [file 41467_2019_13960_MOESM7_ESM.zip › Source_Data/FigureS2/FigureS2B_MotifScanOutput/KmeansCluster1/homerResults/motif9.similar.html]

motif9

## Information for motif9

C
T
A
G
C
T
A
G
T
C
G
A
T
C
G
A
C
T
A
G
C
T
A
G
T
C
G
A
C
T
G
A
C
T
A
G
C
T
A
G
T
C
G
A
T
C
G
A
T
C
A
G
  
Reverse Opposite:  

A
G
T
C
A
G
C
T
A
G
C
T
G
A
T
C
A
G
T
C
G
A
C
T
A
G
C
T
G
A
T
C
G
A
T
C
A
G
C
T
A
G
C
T
G
A
T
C
G
A
T
C
  

|  |  |
| --- | --- |
| p-value: | 1e-102 |
| log p-value: | -2.371e+02 |
| Information Content per bp: | 1.674 |
| Number of Target Sequences with motif | 1070.0 |
| Percentage of Target Sequences with motif | 21.02% |
| Number of Background Sequences with motif | 3955.5 |
| Percentage of Background Sequences with motif | 9.97% |
| Average Position of motif in Targets | 97.2 +/- 50.9bp |
| Average Position of motif in Background | 103.6 +/- 109.7bp |
| Strand Bias (log2 ratio + to - strand density) | -0.2 |
| Multiplicity (# of sites on avg that occur together) | 1.75 |
| Motif File: | file (matrix) reverse opposite |

### Similar de novo motifs found

|  |  |  |  |  |  |  |  |
| --- | --- | --- | --- | --- | --- | --- | --- |
| Rank | Match Score | Redundant Motif | P-value | log P-value | % of Targets | % of Background | Motif file |
| 1 | 0.850 | C T G A C T A G C T A G T C G A C T G A C T A G A C T G T C G A C T G A C T A G C A T G | 1e-51 | -118.326338 | 11.12% | 5.27% | motif file (matrix) |
| 2 | 0.790 | A G C T G T A C A G T C A C G T A G C T A G T C A G T C A C G T | 1e-47 | -108.608776 | 22.61% | 14.44% | motif file (matrix) |
| 3 | 0.848 | G T A C A G T C C G A T C A G T G T A C A G T C A C G T A G C T A G T C G T A C A C G T A C T G T A G C A G T C | 1e-35 | -82.715969 | 5.09% | 1.92% | motif file (matrix) |
| 4 | 0.871 | T C G A C T G A C T A G C T A G T C G A C T G A C T A G C T A G T C G A C T G A C T A G C T A G | 1e-29 | -67.637634 | 6.68% | 3.22% | motif file (matrix) |
| 5 | 0.788 | T A C G C T G A T C G A C T A G C T A G C T A G C T G A C T A G C T A G | 1e-27 | -64.275981 | 10.88% | 6.43% | motif file (matrix) |
| 6 | 0.923 | T G A C G C A T G A T C G T A C G T A C G A C T A G C T A T G C A G T C C G A T A G C T A G T C G T A C | 1e-21 | -50.013799 | 4.05% | 1.78% | motif file (matrix) |
